# Phloem Sap Proteins Are Part of a Core Stress Responsive Proteome Involved in Drought Stress Adjustment

**DOI:** 10.3389/fpls.2021.625224

**Published:** 2021-02-02

**Authors:** Veronica Castañeda, Esther M. González, Stefanie Wienkoop

**Affiliations:** ^1^Department of Sciences, Institute for Multidisciplinary Research in Applied Biology, Universidad Pública de Navarra, Pamplona, Spain; ^2^Unit of Molecular Systems Biology, Department of Functional and Evolution Ecology, University of Vienna, Vienna, Austria

**Keywords:** phloem proteome, drought stress, core stress responsive proteome, redox homeostasis, cell signaling

## Abstract

During moderate drought stress, plants can adjust by changes in the protein profiles of the different organs. Plants transport and modulate extracellular stimuli local and systemically through commonly induced inter- and intracellular reactions. However, most proteins are frequently considered, cell and organelle specific. Hence, while signaling molecules and peptides can travel systemically throughout the whole plant, it is not clear, whether protein isoforms may exist ubiquitously across organs, and what function those may have during drought regulation. By applying shotgun proteomics, we extracted a core proteome of 92 identical protein isoforms, shared ubiquitously amongst several *Medicago truncatula* tissues, including roots, phloem sap, petioles, and leaves. We investigated their relative distribution across the different tissues and their response to moderate drought stress. In addition, we functionally compared this plant core stress responsive proteome with the organ-specific proteomes. Our study revealed plant ubiquitous protein isoforms, mainly related to redox homeostasis and signaling and involved in protein interaction networks across the whole plant. Furthermore, about 90% of these identified core protein isoforms were significantly involved in drought stress response, indicating a crucial role of the core stress responsive proteome (CSRP) in the plant organ cross-communication, important for a long-distance stress-responsive network. Besides, the data allowed for a comprehensive characterization of the phloem proteome, revealing new insights into its function. For instance, CSRP protein levels involved in stress and redox are relatively more abundant in the phloem compared to the other tissues already under control conditions. This suggests a major role of the phloem in stress protection and antioxidant activity enabling the plants metabolic maintenance and rapid response upon moderate stress. We anticipate our study to be a starting point for future investigations of the role of the core plant proteome. Under an evolutionary perspective, CSRP would enable communication of different cells with each other and the environment being crucial for coordinated stress response of multicellular organisms.

## Introduction

Understanding the context in which long-distance signaling function, whole-plant organ and multicellular level processes must be investigated (Lough and Lucas, 2006). In plants, many genes are known to be commonly involved in various environmental stresses, also called plant core environmental stress response (*PCESR*) genes (Hahn et al., 2013). In this work, Hahn and colleagues investigated gene expression profiles of *Arabidopsis thaliana* roots and leaves in response to various abiotic stresses and classified the *PCESR* genes into three different categories: (1) genes regulated in the stressed tissue only (non-systemic), (2) Genes regulated in non-treated tissue only (systemic type I), and (3) Genes regulated in roots and leaves (systemic type II). Overall, *PCESR* genes were found common to several abiotic stresses (Hahn et al., 2013). While gene expression studies enable to distinguish tissue-specific expression profiles, proteomic based approaches can reveal the actual localization and dynamics of proteins and networks, involved in systemic stress response. Hence, more and more studies focus on cell-organelle cross-talk upon stress adaptation using proteomics and metabolomics approaches (Yu et al., 2008; Fürtauer et al., 2019). However, research on cell-to-cell and tissue-to-tissue protein trafficking is still in its infancy. A small number of phloem proteome studies revealed that vascular proteins play crucial roles not only in plant growth and development but also long-distance signaling and stress response (Walz et al., 2004; Aoki et al., 2005). A rapid long distances signal transduction is enabled via the phloem tubes that connects even the most distant organs such as roots and flowers (Kehr and Buhtz, 2008; Nouri and Reinhardt, 2015). Besides photosynthates, the phloem macromolecular translocation system contains small molecules such as phytohormones, small RNA, mRNA and proteins (Lucas et al., 2001; Aoki et al., 2005; Kehr and Buhtz, 2008; Guelette et al., 2012). Those studies disclosed interactions of several proteins or RNA and proteins to facilitate or regulate translocation through the phloem. Furthermore, these phloem proteins were involved in general signaling and stress response mechanism (Walz et al., 2004; Aoki et al., 2005). Moreover, Aoki et al. (2005) demonstrated that phloem long-distance macromolecular trafficking is not only a passive movement but also a controlled, destination-selective process. Their study on phloem proteins from pumpkin (*Cucurbita maxima*) revealed that while the shoot-ward movement was carried passively by bulk flow, while protein-protein interaction (through specific phloem proteins, including eukaryotic initiation factor 5A, and a translationally controlled tumor protein) regulated the selective translocation of RNA binding proteins root-ward.

Previous proteomic studies of *M. truncatula* revealed nodules (Larrainzar et al., 2007, 2009; Gil-Quintana et al., 2015), roots (Staudinger et al., 2012; Lyon et al., 2016; Turetschek et al., 2020) and shoots/leaves (Staudinger et al., 2012, 2016; Lyon et al., 2016) proteins involved in drought stress. However, a functional analysis of ubiquitous proteins, systemically located across distinct plant organs, including petiole and the phloem sap, was still missing. To the best of our knowledge, the role of phloem and petiole proteomes in early drought stress regulation and signaling has also not been investigated thus far.

By applying shotgun proteomics, we extracted a core proteome of 141 proteins, shared ubiquitously amongst several *M. truncatula* tissues, including roots, phloem sap, petioles, and leaves. From those, 92 were individual protein isoforms, systemically present in all investigated plant organs (roots, phloem sap, petioles, and leaves). We compared those with the organ-specific proteomes and found a clear difference in the functional enrichment pattern. The ubiquitous proteome was mainly involved in signaling and redox homeostasis. About 90% of this ubiquitous core proteome, was also significantly responsive to moderate drought stress and connected through a protein–protein interaction network, indicating a global role during environmental stress signaling. These ubiquitous and systemically relevant proteins, therefore, belong to a core environmental stress responsive proteome, which can be clustered in two main groups with changing abundance to moderate drought, either analogously or inversely across the tissues. The data indicate root- as well as shoot-ward long-distance communication through protein regulation or a possible trafficking during drought stress acclimation.

## Materials and Methods

### Plant Growth and Sampling Conditions

*Medicago truncatula* seeds (Jemalong A17) were scarified with sulfuric acid 98% for 7 min washed and then sterilized with 3.5% sodium hypochlorite for 90 s. After a thorough wash, the seeds were soaked in water and left shaking in the dark for 6 h. When the seeds were hydrated, they were transferred to 7‰ agar plates at 4°C for 1 day in the dark, and then incubated at 20°C for 2 days. The seedlings were then planted in pots with perlite:vermiculite (1:3, v/v) and grown for 7 weeks under controlled conditions [22/18°C day/night temperature, 70% relative humidity, 500 μmol m^–2^ s^–1^ (PPF), 12 h photoperiod] and irrigated with Evans medium (Evans, 1981), containing 2.5 mM ammonium nitrate. Water deficit stress was then imposed by water withdrawal and the hydric status of the treatment and control (well-watered) plants was monitored daily by measuring the water potential of the leaves. During the water deficit study, control (C) plants were irrigated with water to avoid nutritional differences, while the water deficit stressed plants were irrigated with 50% of the evapotranspired water every 2 days to induce a progressive and moderate water deficit stress (MD) after 7 days when the leaf water potential (Ψ_*w*_) reached the value Ψ_*w*_ = −1.50 ± 0.02 MPa. At this stage, C and MD plants were harvested, collecting separately, (i) aliquots form the middle part of the root system corresponding to secondary and tertiary roots, (ii) leaflets, separated from the petiole with a scalpel, and (iii) petioles, which were immediately stored at −80°C for further analysis.

### Phloem Sap Exudation

Phloem sap exudation was performed as in Rahmat and Turnbull (2013), with minor modifications. Petioles were cut with razor blades in buffer containing 10 mM EDTA, 10 mM HEPES pH 7. After gently drying the cut with paper, up to 10 leaves per plant were arranged and soaked in Eppendorf tubes containing 1.5 ml of the above mentioned buffer. Phloem sap was collected for 22 h in the dark at 21°C, the leaves were removed and exudate extracts were frozen in liquid nitrogen and stored at −80°C for further use.

### Physiological Parameters

Ψw was measured in the second fully expanded leaf 2 h after the beginning of the photoperiod using a pressure chamber (Soil Moisture Equipment, Santa Barbara, CA, United States) as earlier described (Scholander et al., 1965). Stomatal conductance was measured with a dynamic diffusion porometer (AP4; Delta-T Devices, Cambridge, United Kingdom) in leaflets of the second-fully expanded leaf. Water content was determined in the base to the fresh weight (FW) and the dry weight (DW) obtained after 48 h drying at 70°C using the following formula: WC (%) = (FW-DW)^∗^100/FW. Chlorophyll content was measured in five leaves per plant using a SPAD-502 meter (Konica-Minolta, Japan) in leaflets of the second fully expanded leaf. Measurement of each parameter was carried out on 15 biological replicates for each condition.

### Protein Extraction

Five biological samples of leaves, petioles and roots were used for protein extraction. Aliquots of leaf, petiole (0.07 g), or root (0.5 g) samples were homogenized in a cold mortar with extraction buffer containing 50 mM HEPES pH 7.5, 1 mM EDTA, 20 mM β-mercaptoethanol, 1 mM KCl, 2 mM MgCl_2_, 2.5% PVPP, 1 mM DTT, 1 mM PMSF and 1 proteinase inhibitor cocktail tablet (2 ml of extraction buffer were used for leaf and petiole aliquots and 3 ml for roots). After first centrifugation to remove debris (20.000 *g*, 30 min, 4°C), proteins in the supernatant were precipitated overnight in four volumes of cold acetone (−20°C) and 15 mM NaCl for precipitation optimization (Crowell et al., 2013). Proteins were then pelleted at 10.000 *g*, 4°C, for 10 min.

In the case of the phloem sap exudates, five biological samples per treatment were centrifuged together for debris removal and 1/4 protease inhibitor cocktail tablet was added to each sample. Each biological sample comes from 3 independent phloem sap exudation from a single plant. The phloem sap exudation-buffer was replaced with 50 mM NH_4_HCO_3_ pH 7.5 using Vivaspin 6 MWCO 5000 columns after being pre-rinsed water for Gly contamination removal. After two consecutive centrifugations in a swinging rotor at 3650 *g* (25 min), the protein concentration of the phloem sap exudate was determined by Bradford (1976).

### Protein Digestion

For leaf, petiole and root samples, air-dried protein pellets were dissolved in 250 μL urea buffer containing 7 M urea, 2 M thiourea, 5 mM DTT and 100 mM ammonium bicarbonate (AmBic). After 40 min incubation, protein concentration was determined by Bradford assay, using BSA as a standard. Aliquots containing 50 μg protein were diluted fourfold with trypsin buffer (25 mM AmBic, 10% acetonitrile, 5 mM CaCl_2_) and alkylated for 90 min in the dark (RT) with 10 mM iodoacedamide. Concentrated phloem sap in AmBic buffer volumes corresponding to 50 μg protein was placed in Lo-Bind tubes and DTT, iodoacedamide and CaCl_2_ were added to 10, 15, and 1 mM final concentrations, respectively. Samples were then incubated overnight at 37°C with Porosyzme immobilized trypsin beads (1:20, vol/vol; Applied Biosystems, Darmstadt, Germany). After the addition of formic acid until 1% final concentration, samples were centrifuged in a microfuge at maximum speed for 10 min. Supernatants were desalted with C18-SPEC 96-well plates (Varian, Darmstadt, Germany) according to the manufacturer’s instructions. The eluted peptides were vacuum-dried.

### Nano ESI LC-MS/MS Analysis

Protein digests were dissolved in 2% acetonitrile, 0.1% formic acid and 1 μg of each sample was applied in random order on a C18 column (15 cm × 50 μm column, PepMap^®^RSLC, Thermo scientific, 2 μm particle size) and separated during a 90 min gradient with a flow rate of 300 nL min^–1^ using a UPLC (UltiMate 3000, Thermo Fisher Scientific) for subsequent measurement on an LTQ-Orbitrap Elite (Thermo Fisher Scientific, Bremen, Germany) with the following settings: Full scan range 350–1800 m/z, max. 20 MS2 scans (activation type CID), repeat count 1, repeat duration 30 s, exclusion list size 500, exclusion duration 60 s, charge state screening enabled with the rejection of unassigned and +1 charge states, minimum signal threshold 1000 (Egelhofer et al., 2013).

### Protein Identification and Label-Free Quantification

The canonical *M. truncatula* fasta file was downloaded from UniProt (access date: 6.12.2016). Identification and quantification were performed in MaxQuant v1.5 (Cox and Mann, 2008) with following settings: 20 ppm precursor tolerance for the first peptide search, 8 ppm precursor tolerance for the main search, 0.7 Da match tolerance for ITMS scanned fragment ions; maximum 3 of the following variable modifications allowed per peptide: oxidation of methionine and acetylation of the N-terminus, maximum two missed cleavages allowed, optimum retention time alignment function was determined in a 20 min window, identifications were matched between runs in a 0.7 min window. A minimum of six amino acids was required for peptide identification and at least two peptides were required for protein identification. For label-free quantification (LFQ), one MS2 scan was required with a minimum LFQ ratio of 2. The FDR cut-off for PSM and protein identification was set to a stringent minimum of 1% identifications in the target decoy database (revert). RAW data were also matched against contaminants.

### Statistical Analysis

For the physiological data, fifteen biological replicates were used. Ratios between control and treated samples were considered statistically significant when Student’s *t*-test *p* ≤ 0.05. For proteomics data analysis, three biological replicates were used for phloem sap and five for leaves, roots, and petioles, respectively. Only those protein groups that were found at least in all replicates of one treatment of at least one tissue were used for quantitative analysis. A spectral abundance factor (NSAF) was used for protein abundance normalization according to Zybailov et al. (2008) and Hoehenwarter et al. (2011): protein LFQ values of each identified protein group were divided by their molecular weight and by the sum of all LFQs within a sample. Note: “protein group” is a general term in proteomics, used to indicate that not all proteins are unambiguously identified but members of a group of proteins to which identified peptides match. Hence, only proteins that were unambiguously identified by proteotypic peptides are single isoforms and are specifically indicated in Supplementary Table S2. The statistical significance of protein abundance changes among treatments were evaluated by one-way, non-parametric ANOVA (Kruskal–Wallis). A 10% cut-off was used to remove ANOVA (Kruskal–Wallis) significant proteins with fold changes > 1.4. Kruskal–Wallis test using R (3.5) based InfernoRDN software (v1.1.7234; October 23, 2019), as well as PCA analysis. Missing values were exchanged with half of the minimal value. For enrichment analysis, the protein accessions were uploaded to g:GOSt (organism mtruncatula) from g:Profiler version e99_eg46_p14_f929183^1^ and run as multiquery with default settings (only annotated genes, g:SCS threshold, significance < 0.05). Mapan functional annotations were made using Mercator v3.6 (Lohse et al., 2014) by uploading the uniprot Medicago FASTA file mentioned above. String (v11^2^) was used with the multiple protein search by inputting the protein accessions of the CSRP against *Medicago truncatula* and using default settings with Kmeans Clustering, to gain information on functional protein association networks (Szklarczyk et al., 2019).

## Results

### Physiological Response to Water Deficit Stress in *M. truncatula* Plants

*Medicago truncatula* plants were subjected to water irrigation deficit for 1 week until their leaf water potential reached a moderate water deficit stress (−1.54 ± 0.02 MPa) compared to irrigated control plants (−0.45 ± 0.01 MPa). At this stage, several physiological parameters were determined to assess the effect of this moderate water deficit stress at plant level (Table 1). Substrate water content dropped from 70 to 20% at moderate drought. Both leaf and root water content significantly decreased in response to water deficit stress, with the root tissue presenting higher organ desiccation than the shoot (29 and 5% decrease, respectively). On the other hand, water deficit stressed plants exhibited a total closure of the stomata, while the chlorophyll content remained unaffected (Table 1).

**TABLE 1:** Effect of drought stress on *M. truncatula* plant water status and physiological parameters.

Parameters	Control	Drought
Y_*leaf*_ (MPa)	−0.45 ± 0.01	−1.54 ± 0.02*
Leaf WC (%)	85.24 ± 0.43	81.67 ± 0.43*
Root WC (%)	92.28 ± 0.63	65.52 ± 0.95*
Substrate WC (%)	77.16 ± 0.31	20.88 ± 1.27*
*g*_*s*_ (cm s^–1^)	0.09 ± 0.00	0.00 ± 0.00*
Chlorophyll content	63.04 ± 0.83	63.44 ± 1.74
Shoot (g DW)	1.36 ± 0.06	1.46 ± 0.12
Root (g DW)	2.07 ± 0.11	1.69 ± 0.10

*Values represent the mean ± SE (n = 15). Asterisks indicate significant differences between control and drought (Student’s t-test p < 0.05).*

### Overall Organ Proteome Distribution and Functional Analysis

**FIGURE 1:**
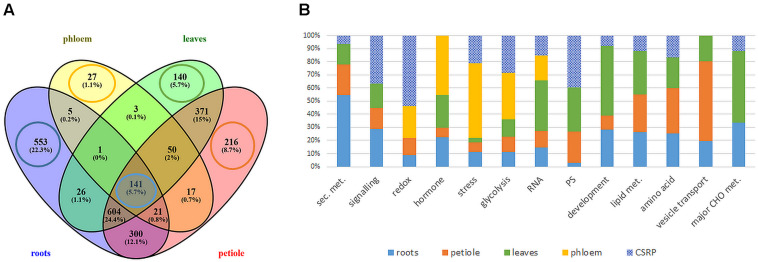
Extraction and functional analysis of tissue specific and core proteins **(A)** comparison of all analyzed tissue proteomes **(B)** relative distribution of protein counts of the functional Mapman categories: tissue specific vs. ubiquitous Core Stress Responsive Proteome (CSRP) (cut-off ≥ 4 proteins of specific proteomes). Here, category “development” is an abbreviation for “cell development,” and “PS” for plastidial proteins.

The MS based proteomics analysis of the leaves, petioles, roots and phloem sap of *M. truncatula* plants resulted in 2696 different protein groups. Peptide information is available at PRIDE repository (Perez-Riverol et al., 2019) with identifier PXD022278. After filtering quantifiable protein groups (see section “Statistical Analysis”), 1651 protein groups were reproducibly found in roots, 1336 in leaves, 1720 in the petiole and 265 in the phloem sap (Supplementary Table S1). Figure 1A shows the distribution of these proteins within the different plant organs: While many of the protein groups were specific to different tissues (22.3% of roots, 5.7% of leaves, 8.7 % of petiole, and 1.1 % of phloem sap) (Figure 1A), more than 50% of the identified proteins were present in more than one organ and 141 (5.7%) common to all investigated plant organs (Figure 1A and Supplementary Table S2). Those core 141 proteins also correspond to 53% of all identified phloem sap proteins. Of these 141 ubiquitous proteins, most, (92; 65%) were identified based on prototypic peptides exclusively and thus referring to unique unambiguously identified protein isoforms (no groups) (Supplementary Table S2). Noteworthy, the largest common group of 604 proteins (24%) excludes the phloem sap (Figure 1A) and will not further be analyzed in this study. Here, we focus on the 141 core proteins and compare them to the very organ-specific proteins, solely.

#### Organ/Tissue-Specific Proteomes

The GO analysis of the organ-specific proteomes showed the enrichment of proteins mainly of the cytoplasm for roots, petioles and also leaves (more so chloroplasts) while the phloem was enriched mainly in the extracellular region (Table 2, Supplementary Figure S1, and Supplementary Table S1). Main molecular functions associated to each organ are “catalytic activity” for roots, “peroxidase activity” for phloem sap, “chlorophyll-binding” for petiole and “acylgycerol lipase activity” for leaves (Table 2 and Supplementary Table S1). A Mapman functional analysis of the organ-specific proteomes revealed 14 major categories (≥4 proteins) (Figure 1). Taken aside the overall most dominant category protein regulation (not enriched), the next largest categories were secondary metabolism, signaling, stress, amino acid, hormone, RNA, lipid metabolism, cell development, major CHO metabolism, redox, cell vesicle transport, glycolysis, and plastidial (PS) metabolism (Figure 1B). The specific distribution of those protein categories along the organs revealed particular differences in the relative abundances (Figure 1B). The categories stress, hormone, glycolysis and redox were relatively most abundant in the phloem sap; secondary metabolism, major CHO metabolism and signaling were enriched in roots; plastidial (PS), cell development and major CHO metabolism proteins showed highest relative abundance in leaves and cell vesicle transport and amino acid-related proteins were most abundant in the petioles.

**TABLE 2:**
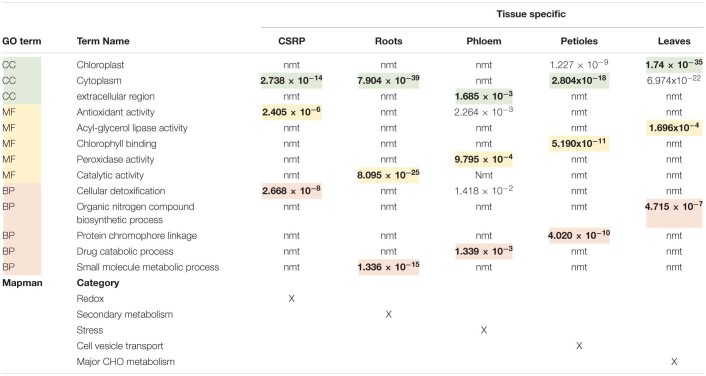
Overview of the core stress response proteome (CSPR) and the tissue specific protein (only found in one specific tissue) GO functional term enrichments and corresponding P.adj-values extracted from Supplementary Figure S1, as well as the major Mapman categories taken from Figure 1B.

*CC, cellular compartment; MF, molecular function; BP, biological process. nmt, not major (top 10) GO term for this tissue.*

#### Core Proteome and Relative Distribution

**FIGURE 2:**
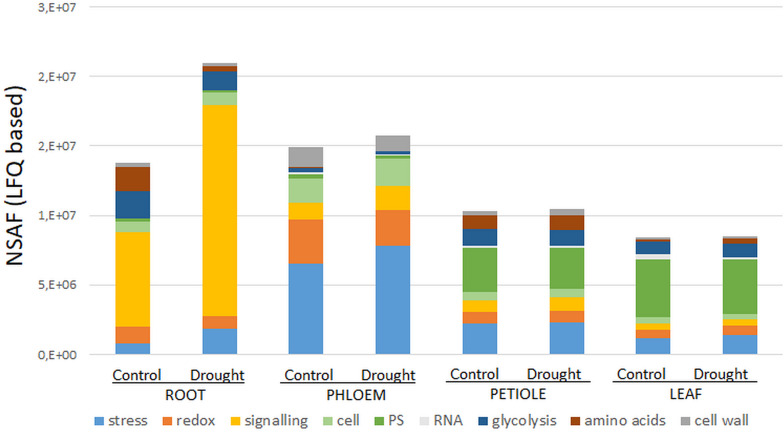
Major Mapman categories of the ubiquitous core stress responsive proteome (CSRP) and their relative abundance distribution (sum of normalized LFQ values of categories cut-off ≥ 4 proteins) comparing controls to moderate drought stress across the tissues. Here, category “cell” is an abbreviation for “cell organization,” and “PS” for photosynthesis proteins.

Secondary- hormone-, lipid- and major CHO metabolism, as well as cell development and cell vesicle transport categories, were not amongst the major groups of the ubiquitous (core) proteins (<4 proteins) (Figure 1B). In contrast to the organ-specific proteomes, the core proteins were further enhanced in redox and signaling as well as plastidial (PS) proteins (Figure 1B). Altogether, we found nine major functional categories (≥4 proteins) for the core plant proteome composed mainly by stress and redox > signaling > cell organization (cell) and plastidial (PS) > glycolysis > amino acids and cell wall proteins > RNA (Figure 2 and Supplementary Table S2). To decipher the relative protein abundance distribution of this core proteome of identical protein isoforms, ubiquitously identified across the specific organs, further, a closer organ distribution analysis based on spectral abundance (LFQ based summed NSAF) of the major Mapman categories of the core proteome was performed (Figure 2). These revealed differences in abundance of the core proteins, among the organs: the roots were again dominated by signaling proteins (mostly G-proteins and receptor kinases like for the specific proteome) but additionally also by glycolysis and amino acid metabolism proteins. Relatively the most enhanced core protein of the roots compared to the other organs was P93333, a PR-10 receptor kinase, possibly involved in ABA binding. It was 121-fold more abundant in roots compared to petioles, 22-fold higher than in the phloem and 84-fold more abundant than in the leaves (Supplementary Table S2).

The phloem sap core proteins were similar to the phloem specific proteins, enhanced in redox and stress related proteins but also by cell organization related proteins (Figure 2). However, lipid metabolism related Acyl-CoA-binding domain protein (G7K6T1, 120-fold more abundant than in leaves, 50-fold more abundant compared to petioles and 33-fold higher than in roots) was the most abundant phloem core protein amongst others (see also highest loadings PC1, Supplementary Table S2).

Petioles showed higher levels of the amino acid metabolism related proteins while leaves showed higher abundance levels of those core proteins of assigned to plastids and RNA categories. The GO enrichment analysis further revealed that cytoplasm proteins involved in the cellular response to oxidative stress and antioxidant activity and cellular detoxification were the major functional categories (Table 2 and Supplementary Figure S1e).

### Functional Analysis of the Core Proteins in Mild Drought Stress Regulation

Out of the 141 core proteins, 126 (89%) changed significantly in at least one organ when plants were subjected to moderate water deficit stress (*p* ≤ 0.05 and a fold change ≥ 1.25) (Supplementary Table S2). The root core proteome showed the largest number of significantly changed proteins (96; 48 down, 48 up), followed by the phloem sap core proteome (51; 36 down, 14 up), the leaves (36; 11 down, 25 up) and the petioles (17; 11 down, 6 up) (Supplementary Table S2). Compared to Figure 1B, Figure 2 allows for the relative abundance comparison based on signal intensity (LFQ) rather than protein numbers alone. Hence, Figure 2 enables to compare between control and stress treated protein abundances. It reveals a general increase of stress and signaling proteins, such as receptor kinases, in roots and phloem sap during drought, while redox related protein abundances decrease. Additionally, a reduction of amino acid and glycolysis related protein levels upon drought are visible, but only in roots (Figure 2).

**FIGURE 3:**
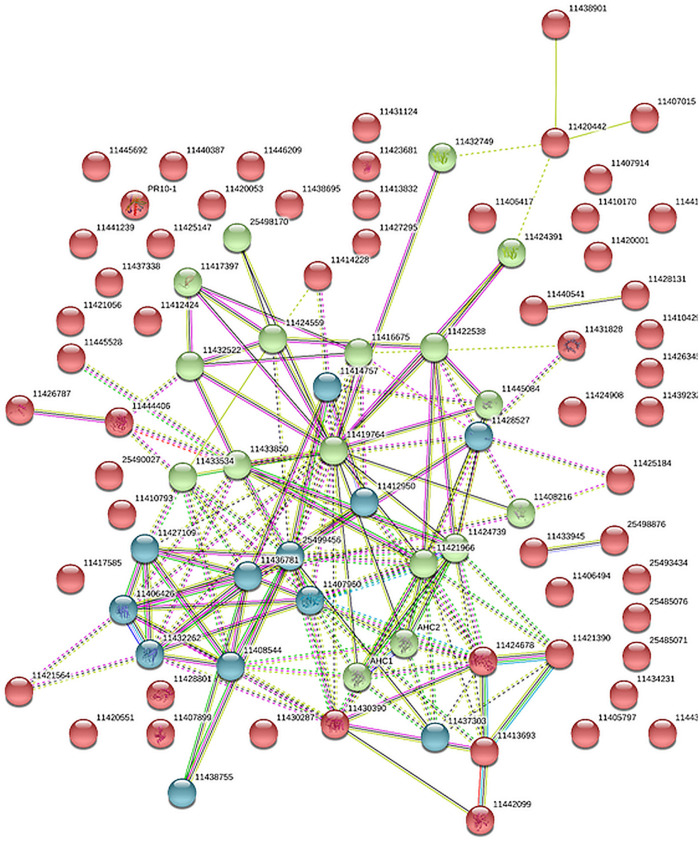
Protein association network analysis of the core stress response proteome (CSRP) using STRING. Colors (red, green cyan) are based on a kmeans cluster analysis (see also Supplementary Table S2).

The protein interaction analysis referenced a strong protein network of clear biological connectivity (PPI enrichment *p*-value: < 1.0e-16). Three main network clusters (kmeans) that include redox enriched proteins in the cyan string cluster, carbon and nitrogen metabolism-related proteins, enriched in the green cluster, as well as a red cluster mainly composed of cell wall and known stress response proteins, were depicted (Figure 3 and Supplementary Table S2).

## Discussion

### The Core Stress Responsive Proteome Differs From Tissue-Specific Proteomes and Mainly Consists of Proteins Involved in Redox Homeostasis and Signaling

We extracted a set of ubiquitous proteins present in all investigated plant tissues, responsive to drought, which we call the core stress responsive proteome (CSRP), respectively, and compared this to the tissue-specific proteomes to understand the distinct function of the CSRP. However, it remains unclear, whether these CSR proteins are translated simultaneously in the various cell tissues or individually in organ-specific cells and then transduced throughout the whole plant. Hahn et al. (2013) revealed that most *PCESR* genes have paralogs and proposed a backup function of presumably redundant information of incoming stresses signals. Here, most of the proteins of the CSRP were root and phloem enhanced, meaning that these initial protein levels were relatively more abundant within the root and/or phloem proteomes compared to the proteome of the other tissues (Figure 2). The CSR proteins of roots and phloem sap were also the most responsive tissues to drought in terms of numbers of proteins and abundance levels (normalized LFQs), indicating enhanced gene expression of these proteins in roots and/or phloem sap under well-watered as well as stress conditions. Major functional groups of the CSPR were those involved in redox and signaling, and also more abundant compared to tissue-specific functions (Figures 1, 2), revealing a crucial role of protein pathways involved in rapid communication and regulation. Remarkably, six out of the seventeen redox related proteins of the CSRP are included in the protein association network (Figure 3), supportive for a controlled interaction of these proteins maintaining metabolism under optimal growth conditions and suggesting a major function of the CSRP apparatus in systemically regulating and maintaining redox-homeostasis.

**FIGURE 4:**
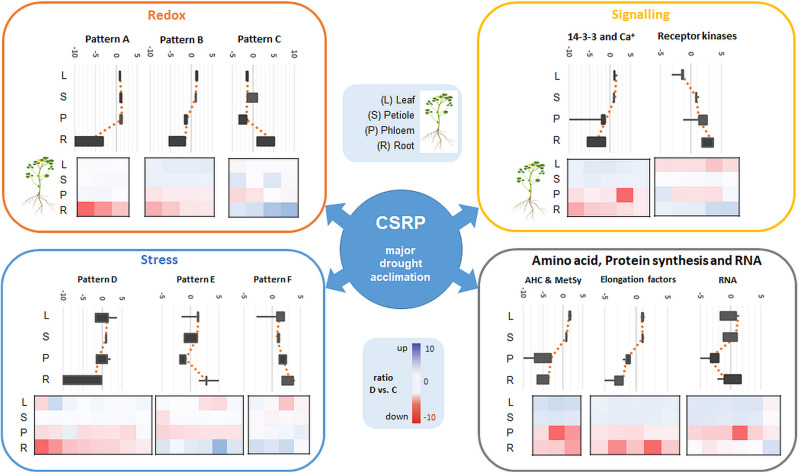
Overview of the major Mapman categories (redox, signaling, stress as well as amino acid, protein synthesis and RNA) of the ubiquitous CSRP and patterns of overall relative changes in protein abundance as a response to moderate drought stress in the different tissues (sum of normalized LFQ values of categories cut-off > 4 proteins). Diagrams show main visual patterns of summed ratios of the functional protein groups listed in Supplementary Table S2. **Upper Box-plot** diagrams show the distribution of the relative changes (D vs. C) of all proteins belonging to a characteristic pattern along the different tissues. **Lower plots** are heatmaps of D/C ratios of individual proteins for the different tissues grouped into the different patterns and categories. Proteins included in each pattern and category are referenced in Supplementary Table S2.

Furthermore, proteins involved in translation (synthesis and degradation) are the largest amongst all annotated proteins here and in most other proteomic studies and therefore not specifically enriched (Walz et al., 2004; Aoki et al., 2005). Nevertheless, our data confirm a crucial role of parts of the translational apparatus within the CSRP, possibly mobile and therefore interesting for future research (Figure 4, elongation factors).

### Most of the CSRP Is Involved in Moderate Drought Stress Adjustment

Regarding the model legume *M. truncatula*, the water potential exhibited by the water deficit stressed plants (−1.5 MPa) might be considered just around the limit of the permanent wilting point, an energy state below which the extraction of water from the soil solution is generally considered to be inhibited and commonly classified as moderate drought stress (Fitter and Hay, 2002). Indeed, the stress applied in the present study (−1.5 MPa) did not significantly modify the chlorophyll content (Table 1). The regulation of leaf stomatal conductance is crucial in plants as it is vital for the prevention of desiccation (Dodd, 2013). Hence, we consider this as an early phase of plant drought stress just at the limit of a functional phloem able to maintain trafficking of molecules essential for a possible systemic acclimation of stress. Hydraulic signal communication of the water status between root and shoot is crucial in order to sustain plant growth and photosynthetic activity in an environment of limiting or changing water availability (Christmann et al., 2007).

Most proteins of the CSRP (98%) responded significantly to moderate drought stress. Within these, all redox proteins show significant changes at least in one of the four tested tissues during drought (Supplementary Table S2). Redox homeostasis and antioxidant activities are assigned as major regulation mechanisms, strongly correlating with drought (Laxa et al., 2019). *M. truncatula* (Jemalong A17), showed antioxidant responses in leaves and roots already under moderate drought (Filippou et al., 2011). Since plant accession was the same and treatments were similar to ours, we assume comparable ROS stress levels in the present study.

The redox apparatus of the CSRP was abundant in all organs analyzed. Interestingly, however, individual protein isoforms were differently intense or even inversely responsive to early stress in those various tissues (Figure 4). Proteins involved in redox homeostasis, such as peroxidases function in signal triggered stress responses linked to ABA, Ca^2+^ fluxes and sugar sensing (Cruz De Carvalho, 2008). During drought response, they have been shown to work both upstream and downstream of the ABA-dependent signaling pathways. Here, we found several of the core redox proteins that were regulated opposite upon early drought stress between roots and leaves. Amongst those, three proteotypic isoforms, thioredoxins (A0A072V2Y4 and G7IBZ4), and a monodehydroascorbate reductase (A0A072VJS5) were significantly depleted in roots while accumulated or not affected in the leaves. Hence, these proteins either could belong to a paralogs *PCESR* gene regulation or involved in long-distance protein translocation in order to regulate the global plant redox homeostasis at a moderate state of drought stress from root to shoot. A similar pattern was found for the proteins of the amino acid metabolism, where, e.g., methionine synthase (G7L0I7) and two adenosylhomocysteinase (AHC) isoforms (Q84RD8 and Q84RD9) were significantly reduced in roots and phloem but accumulated in leaves. These key enzymes of the *S*-adenosylmethionine cycle are known to play major roles in methylation regulation of DNA, proteins, and other metabolites (Li et al., 2015). By controlling the intracellular concentration of adenosylhomocysteine, AHCs maintain the cellular methylation potential (Li et al., 2015). Our network analysis (Figure 3) additionally demonstrates a clear crosslink of the two AHC isoforms with the redox cluster providing evidence for a connection between the methionine cycle and redox signaling. This is not surprising as methionine is a known target for oxidation linking the two functional categories with the regulatory role of reversible post-translational modification during stress (Hoshi and Heinemann, 2001). Hence, our data suggest a regulatory network, where a set of specific proteins involved in methylation and oxidation is transferred from roots to leaves via the phloem sap during moderate drought. This scenario seems more likely than an opposite translational regulation – degradation in roots and synthesis in leaves- of the same proteins in different tissues at the same time and in response to the same stress, which would not be in line with the backup function hypothesis proposed by Hahn et al. (2013). A third possibility might be a dynamic protein translational apparatus that is translocated to the area of need as the phloem is known to transduce large messenger RNA (Kehr and Buhtz, 2008; Kehr and Kragler, 2018). In line with this hypothesis, three out of the six proteins of the CSRP participating in RNA regulation were RNA binding proteins. Since the possible role of mobile RNAs in regulating transcription and translation across the plant has been extensively discussed, the presence of RNA regulating proteins seems congruous.

Overall, in roots, proteins involved in degradation regulation were significantly increasing during drought. In contrast, protein levels of the synthesis apparatus decreased in roots while simultaneously increasing in the leaves. This pattern correlates positively with the above described response pattern of the redox and amino acid metabolism. Protein turnover is known to increase during and after drought exposure, crucial for stress adjustment and recovery (Henckel, 1970; Lyon et al., 2016).

In contrast, the second-largest functional category of the CSRP, signaling, which consists mostly of ABA activated signaling pathway proteins, was mainly accumulated in roots while depleted in the leaves upon moderate drought (Figure 4). In this case, the overall increase in protein abundance of the signaling proteins in roots and phloem sap (ABA-responsive proteins G7INB7 and G7IMZ3), indicates protein *de novo* synthesized rather than trafficking from shoots to roots upon stress. This is particular interesting as ABA is usually known to be transported via the transpiration stream from roots to leaves, to communicate the soil water status, causing stomatal guard cell closure at the very initial stage of osmotic stress (Christmann et al., 2007). Hence, our results indicate an enhanced activity of proteins, particularly involved in ABA binding in both, the roots and the phloem during drought but opposite to the transpiration stream, depleting in leaves (Figure 4 and Supplementary Table S2). At the same time, several proteins involved in the ABA response and biosynthesis such as a Noduline-like protein (G7JGC9) are also increased most significantly in roots gradually less toward the leaves during moderate drought. This discloses a systemically reconciled ABA production but no clear translocation mechanism across the plant conducted by the CSRP at this stage of drought stress. Nevertheless, the CSRP function in ABA signaling is further supported by the presence of three distinct protein isoforms of Fasciclin-like arabinogalactan (FLA). These FLA proteins belong to a multigene family of cell adhesion molecules (Johnson et al., 2003; Seifert et al., 2014). These authors showed that *Arabidopsis FLA* genes interact with ABA and control root growth and abiotic stress. During drought, our data reveal that these isoforms are acting antagonistically. Two isoforms (G7K503 and G7K0M1) significantly (*p*<0.05) decrease in the roots while one putative FLA protein isoform (A0A072U0Q0) reacts by significantly (*p*<0.05) accumulating in roots and leaves. Isoforms A0A072U0Q0 and G7K0M1 are possibly also interacting with each other (Figure 3). Since FLAs are linked to the cell wall, these particular CSRP isoforms seem therefore involved in systemic cell wall and growth regulation across the whole plant.

A common and early response to drought stress is usually the perturbation of photosynthetic activity (Aranjuelo et al., 2011). As we focus on the CSRP in response to drought, we just find seven proteins putatively related to photosynthesis. However, only Ribose-5-phosphate isomerase (G7L1U4) seems significantly reduced in the leaves upon drought otherwise most are significantly reduced or even increased in the roots, suggesting that these proteins are most likely not directly involved in photosynthetic activity. It would be possible to test the specific proteomes or the total proteome of the leaves in this study as well but it was not the aim of this study to repeat previous research.

### More Than 50% of the Phloem Proteome Is Connected to the Core Stress Responsive Proteome, Involved in the Control of Drought Stress

Here, we identified 265 phloem sap proteins of *M. truncatula*, allowing focusing on the subset of the CSRP and linking the different tissues during drought stress. In comparison, Guelette et al. (2012) investigated the largest proteomic study of the phloem exudate of *Arabidopsis thaliana*, where 65 proteins were found. In our study, more than half (53%) of the phloem proteins belong to the CSRP. While ‘stress’ was not the large protein category within the CSRP (Figure 1B), it was largest within the phloem sap proteins in terms of number and overall protein abundance (Figures 1B, 2). Besides protein regulation, it was also the most responsive category of the phloem sap, upon moderate drought stress (Supplementary Table S2). Notably, due to the strong linkage with the CSRP, and also because phloem sap proteins that are not belonging to the CSRP are not responsive to moderate drought, it is not possible to fully separate discussion of phloem sap function from the CSRP. Hence, this finding may supports the important function of the phloem in long-distance stress regulation.

Furthermore, we found several pathogen resistant (PR) proteins to be within the stress category proteins (Supplementary Table S1). Amongst those, the largest number (7) were chitinases (class 4 PR proteins) of which three (G7ID31, G7LA76 and A0A072UQU5) were also found in the CSRP (Supplementary Table S2). Similarly, PR proteins have previously been described in phloem exudates (Giavalisco et al., 2006; Lin et al., 2009). Additionally, several heat shock and LEA proteins were also comprised of the stress category of the CSRP (Supplementary Table S2). Since most of these proteins responded significantly to drought, they appear to be multi-functional and not restricted to pathogen defense, and heat stress. LEA proteins, for instance, have already been recognized to respond to drought but also pathogen attack (Ranjbar Sistani et al., 2017). Furthermore, heat shock and LEA proteins are functioning as chaperones, regulation protein–protein interactions such as protein aggregations and the protein structural integrity. They have been suggested to play an important role in the regulation of protein transport through sieve elements (Aoki et al., 2002, 2005). Nevertheless, their overall abundance increases during drought conditions in phloem sap and roots. These data indicate that they are initially mainly synthesized in the phloem and slightly upon stress but possible partially translocated toward the roots, where their overall levels also increased during drought (Figure 2). The high abundance levels of these proteins in the phloem sap may also suggest that they are actively needed, locally. It should be noted, that the enhanced levels of stress-, as well as redox-proteins (peroxidase activity related) (Figure 2) of the phloem sap, might also be affected by the long extraction time of the phloem sap. However, Gaupels et al. (2017) also support systemic propagation of redox signaling through the phloem. Noticeably, glutaredoxin (G7IID), connected to the redox homeostasis network (Figure 3), showed the most significant accumulation of all enzymes in the phloem sap upon drought. This indicates a phloem sap specific induction of electron transfer- and protein disulfide oxidoreductase activity regulation, during drought stress.

The presence of glycolysis metabolic proteins in the CSRP indicates that this pathway is systemically present and regulated across the plant. This is not surprising as it is a key metabolic pathway, involved in cellular energy maintenance. During drought, they were most significantly changing in the roots (depleting) but also in the phloem, indicating a local breakdown in energy maintenance (Supplementary Table S2). Interestingly, two triose-phosphate isomerases (TPI) (I3S3S0 and A0A072U2W1) decreased significantly in phloem sap and roots. TPI is a key enzyme of the glycolytic pathway and its activity is known to decrease under drought stress (Sharma et al., 2012). Its role within the CSRP and in a possible modulation of the plant carbon sink-source relation during drought is not clear. However, sucrose has been shown to be metabolized via glycolysis in the phloem of *Ricinus communis* L. seedlings (Geigenberger et al., 1993) and mainly in the companion cells (van Bel, 2003).

The cellular component analysis of the mapman protein category photosynthesis, actually showed the cytosol as major localization, indicating that these proteins of the CSRP are nuclear-encoded. Surprisingly, a Rubisco large subunit protein group (G7JG19/S4T017) was detected suggesting a possible contamination from the leaf samples. However, the most abundant Rubisco large subunit isoform detected in the leaves and not detected in the phloem was (B7FHB2, twofold more abundant than the group G7JG19/S4T017). Hence, a carry over from leaf samples seems not a likely explanation. Previously, a set of ‘plastidial’ proteins have also been detected in phloem exudates of *Arabidopsis* (Guelette et al., 2012). They discussed the possibility of active transport of these proteins into the sieve element from companion cells through plasmodesmata. Role and presence, however, remain speculative at this point.

The Mapman category ‘cell’ comprises three subcategories, -cycle, -organization and -development. The cell-cycle proteins of the CSRP are composed of three peptidylprolyl isomerase (PPIase) isoforms (Supplementary Table S2) (I3S3K3, G7JPK2, A0A072VNE4). PPIases are ubiquitous plant proteins, involved in protein unfolding and cyclosporine A binding and described to be important abiotic stress regulators induced by ABA (Sharma and Singh, 2003). Indeed, we found them significantly increased in roots and partly, in the phloem during drought conditions while depleted in the leaves (I3S3K3, G7JPK2, A0A072VNE4) positively correlating with the ABA-regulation proteins (Supplementary Table S2). The protein association network study indicates a strong interaction of PPIase isoform, G7JPK2, with four other CSR proteins, positively correlating with an ubiquitin (G7K8J5), and a heat shock protein (Q1SKX2), and negatively correlating with a peptide methionine sulfoxide reductase (G7K8J3), and the nascent protein-associated complex alpha chain protein (G7L4T7) (Figure 3). It indicates that this PPIase reconciles protein translation processes in roots during drought. Since PPIases interact selectively and non-covalently with cyclosporin A, within the CSRP, these might function in translocation of this immune repressive cyclic peptide across the plant.

Among the cell organization related proteins within the CSRP actin-related and -binding proteins such as profilin, villin, fibrillin, cofilin, are mainly enriched in the phloem sap. Cell structural components, such as actin and profilin, have been found in several phloem exudate studies (Staiger et al., 1997; Rodriguez-Medina et al., 2011). Actin and profilin were described to be constantly delivered into the sieve elements, trafficking through the translocation stream of *Ricinus* (Schobert et al., 2000). Hence, the ubiquitous presence of this kind of proteins in all the plant organs indicate that there is a dynamic and systemic network of cytoskeletal elements involved in remodeling cytoplasmic architecture, which is also responsive to environmental stress. Under drought stress, fibrilin (G7KCE7) decreased in the phloem while increasing in the root and cofilin (G7IFU0) showed the opposite pattern, suggesting a complex modulation of these cytoskeletal elements.

Guelette et al. (2012) proposed lipid metabolism to be an important feature of the phloem metabolism in *Arabidopsis*. Here, only one lipid metabolism protein, an acyl-CoA binding protein (G7K6T1) was part of the CSRP and enhanced in the phloem sap (highest loadings PC1), and three lipid transfer proteins were exclusively identified in the phloem sap. Hence, lipid metabolism seems to some extent to be involved in phloem function, but at least in *Medicago*, it is only a minor functional protein group, which does not make it less interesting.

It should be noted that a larger set of common proteins was found when excluding the phloem sap proteome, indicating that these proteins either were of very low abundance in the phloem sap or not present. This, however, also means that focusing on ubiquitous proteins including the phloem sap, increases the probability that proteins of the CSRP are actually involved in translocation. Nevertheless, further studies are necessary to understand the interaction network of the CSRP fully. We also want to mention that other mechanisms related to signal transduction through the xylem upon drought seem relevant. A previous study of maize (Alvarez et al., 2008), demonstrated changes in secondary metabolites such as ABA and cytokinin. On the level of proteins, they, however, only found peroxidases, suggesting that the xylem sap proteome is probably not associated to the here described CSRP.

## Conclusion

**FIGURE 5:**
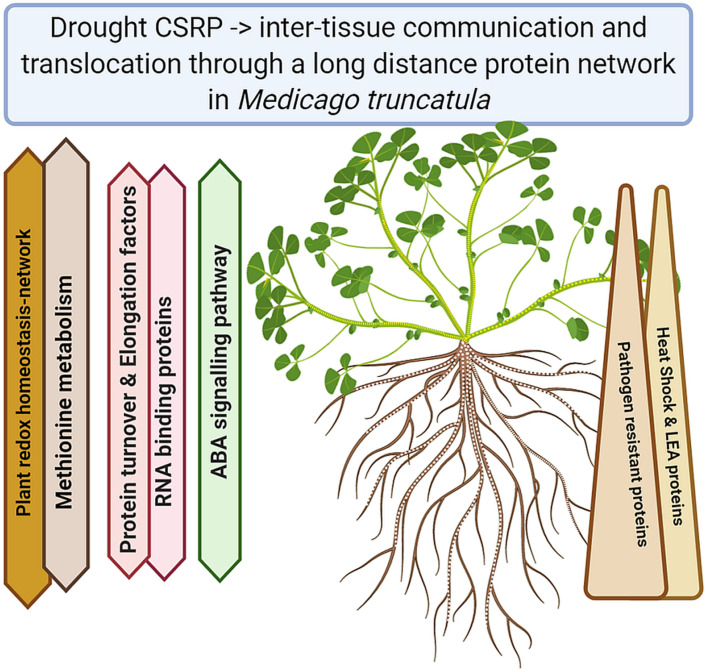
Summary of the major functional Core Stress Responsive protein changes upon drought stress (Created with BioRender.com).

Understanding the plant stress and signaling networks is of major concern when it comes to stress adjustments and ultimately increased plant tolerance. In the last two decades, research has shown that there is more to the phloem then just a transport system, dependent on the metabolism of leaves and roots. Our study demonstrates the existence of a core proteome in *Medicago truncatula*, across different tissues involving the phloem (Figure 5). The proteomic data reveal a crucial role of the phloem in drought stress regulation through a system of core stress responsive proteins (CSRP) present throughout the whole plant. The CSRP, protein isoforms found simultaneously across all tested plant organs, is headed by major stress related functions, possibly networks, involved in redox homeostasis and signaling. Besides it multiple roles, we present evidence that the phloem’s specific proteome function is led by stress response proteins while within the CSRP it functions as a systemic redox signaling translocator, linking below and above ground communication of the plant in order to fine-tune stress response. This is an initial view on core proteins that may or may not exist in other plants and may have their evolutionary origin in enabling communication of different cells with each other and the environment, making it an interesting field to study.

## Data Availability Statement

The datasets presented in this study can be found in online repositories. The names of the repository/repositories and accession number(s) can be found in the article/Supplementary Material.

## Author Contributions

EG and SW conceived the experiment and research focus. VC performed the experiment and analyzed the physiological and proteomic data. SW contributed to the mass spectrometry and proteomics datamining, VC and SW wrote the manuscript. EG revised the manuscript. All the authors contributed to the article and approved the submitted version.

## Conflict of Interest

The authors declare that the research was conducted in the absence of any commercial or financial relationships that could be construed as a potential conflict of interest.

## Footnotes

1 https://biit.cs.ut.ee/gprofiler/gost

2 https://string-db.org/
