# Supplementary material for: Phloem Sap Proteins Are Part of a Core Stress Responsive Proteome Involved in Drought Stress Adjustment
Source: Front Plant Sci. 2021 Feb 2;12:625224. doi: 10.3389/fpls.2021.625224 (PMC7884324; doi:10.3389/fpls.2021.625224)
Supplement: Supplementary file 1 [file Data_Sheet_1.PDF]

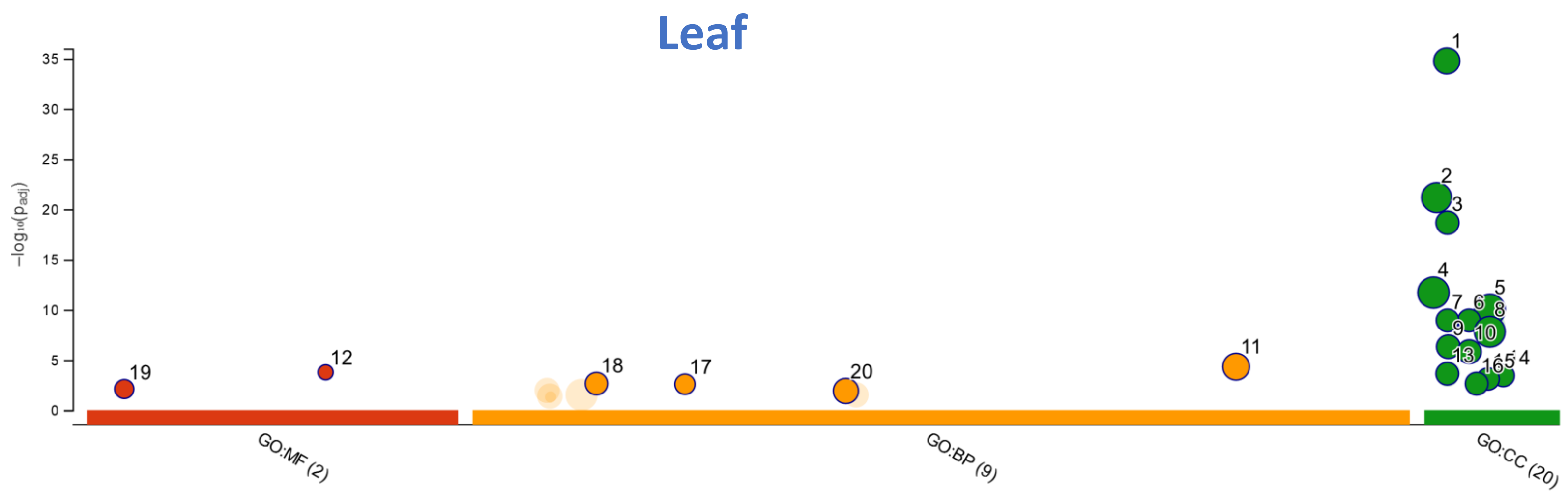

ID	Source	Term ...	Term Name	p_{adj} (query_1)
1	GO:CC	GO:0009507	chloroplast	1.747×10^{-35}
2	GO:CC	GO:0005737	cytoplasm	6.974×10^{-22}
3	GO:CC	GO:0009532	plastid stroma	2.197×10^{-19}
4	GO:CC	GO:0005622	intracellular	1.965×10^{-12}
5	GO:CC	GO:0043229	intracellular organelle	9.708×10^{-11}
6	GO:CC	GO:0031976	plastid thylakoid	1.154×10^{-9}
7	GO:CC	GO:0009534	chloroplast thylakoid	1.154×10^{-9}
8	GO:CC	GO:0043227	membrane-bounded organelle	1.567×10^{-8}
9	GO:CC	GO:0009579	thylakoid	4.786×10^{-7}
10	GO:CC	GO:0031984	organelle subcompartment	1.511×10^{-6}
11	GO:BP	GO:1901566	organonitrogen compound biosynthetic process	4.716×10^{-5}
12	GO:MF	GO:0047372	acylglycerol lipase activity	1.696×10^{-4}
13	GO:CC	GO:0009526	plastid envelope	2.354×10^{-4}
14	GO:CC	GO:0055035	plastid thylakoid membrane	3.655×10^{-4}
15	GO:CC	GO:0042651	thylakoid membrane	7.832×10^{-4}
16	GO:CC	GO:0034357	photosynthetic membrane	2.293×10^{-3}
17	GO:BP	GO:0019684	photosynthesis, light reaction	2.603×10^{-3}
18	GO:BP	GO:0009657	plastid organization	2.250×10^{-3}
19	GO:MF	GO:0004620	phospholipase activity	7.816×10^{-3}
20	GO:BP	GO:0043043	peptide biosynthetic process	1.218×10^{-2}

**version**	e99_eg46_p14_f929183
**date**	14.5.2020, 16:19:23
**organism**	mtruncatula

g:Profiler

Figure S1a. GO enrichment analysis (g:Profiler) of the specific leaf proteome.

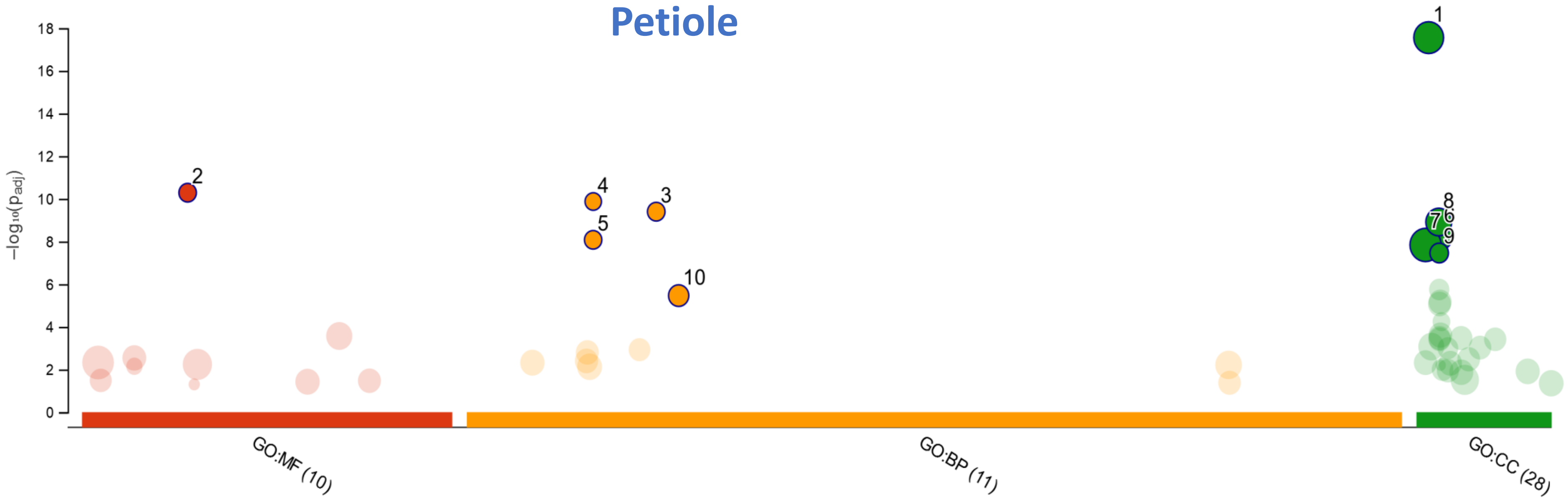

ID	Source	Term ...	Term Name	p_{adj} (query_1)
1	GO:CC	GO:0005737	cytoplasm	2.804×10^{-18}
2	GO:MF	GO:0016168	chlorophyll binding	5.190×10^{-11}
3	GO:BP	GO:0018298	protein-chromophore linkage	4.020×10^{-10}
4	GO:BP	GO:0009768	photosynthesis, light harvesting in photosystem I	1.343×10^{-10}
5	GO:BP	GO:0009765	photosynthesis, light harvesting	8.388×10^{-9}
6	GO:CC	GO:0009536	plastid	5.689×10^{-9}
7	GO:CC	GO:0005622	intracellular	1.456×10^{-8}
8	GO:CC	GO:0009507	chloroplast	1.227×10^{-9}
9	GO:CC	GO:0009522	photosystem I	3.484×10^{-8}
10	GO:BP	GO:0019684	photosynthesis, light reaction	3.445×10^{-6}

version e99_eg46_p14_f929183
date 14.5.2020, 16:07:23
organism mtruncatula

g:Profiler

Figure S1b. GO enrichment analysis (g:Profiler) of the specific petiole proteome.

Phloem

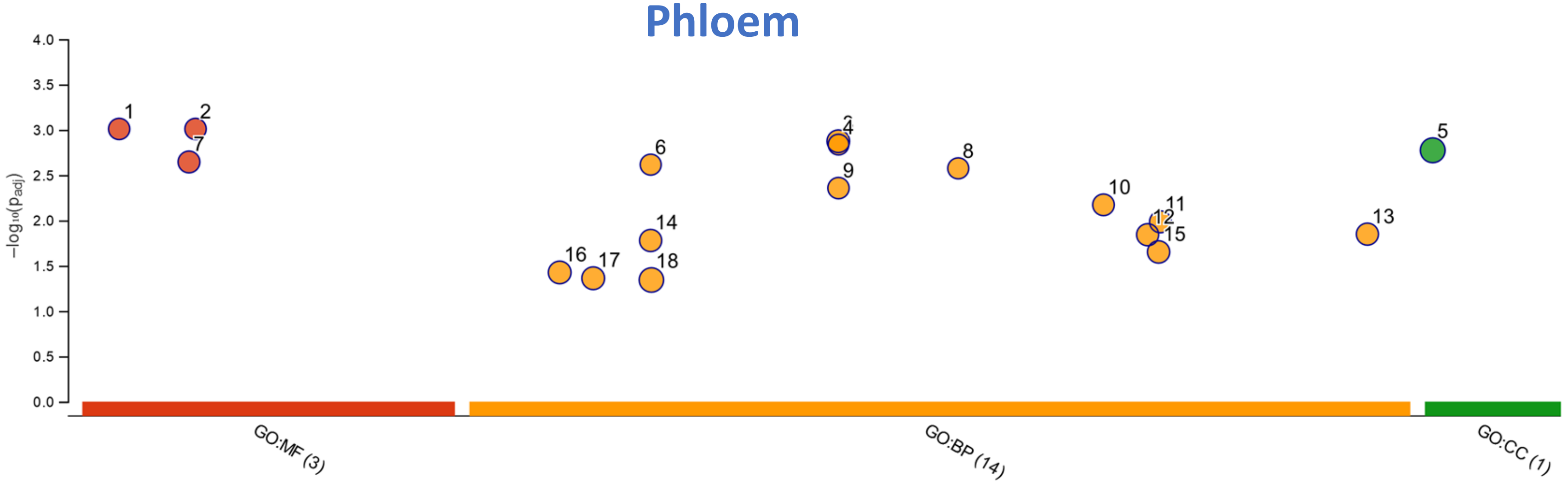

ID	Source	Term ...	Term Name	p_{adj} (query_1)
1	GO:MF	GO:0004601	peroxidase activity	9.795×10^{-4}
2	GO:MF	GO:0016684	oxidoreductase activity, acting on peroxide as acc...	9.795×10^{-4}
3	GO:BP	GO:0042737	drug catabolic process	1.339×10^{-3}
4	GO:BP	GO:0042744	hydrogen peroxide catabolic process	1.457×10^{-3}
5	GO:CC	GO:0005576	extracellular region	1.685×10^{-3}
6	GO:BP	GO:0017001	antibiotic catabolic process	2.426×10^{-3}
7	GO:MF	GO:0016209	antioxidant activity	2.264×10^{-3}
8	GO:BP	GO:0051187	cofactor catabolic process	2.666×10^{-3}
9	GO:BP	GO:0042743	hydrogen peroxide metabolic process	4.401×10^{-3}
10	GO:BP	GO:0072593	reactive oxygen species metabolic process	6.733×10^{-3}
11	GO:BP	GO:0098869	cellular oxidant detoxification	1.037×10^{-2}
12	GO:BP	GO:0097237	cellular response to toxic substance	1.439×10^{-2}
13	GO:BP	GO:1990748	cellular detoxification	1.418×10^{-2}
14	GO:BP	GO:0016999	antibiotic metabolic process	1.667×10^{-2}
15	GO:BP	GO:0098754	detoxification	2.229×10^{-2}
16	GO:BP	GO:0006979	response to oxidative stress	3.754×10^{-2}
17	GO:BP	GO:0009636	response to toxic substance	4.351×10^{-2}
18	GO:BP	GO:0017144	drug metabolic process	4.545×10^{-2}

**version**	e99_eg46_p14_f929183
**date**	14.5.2020, 16:15:37
**organism**	mtruncatula

g:Profiler

Figure S1c. GO enrichment analysis (g:Profiler) of the specific phloem sap proteome.

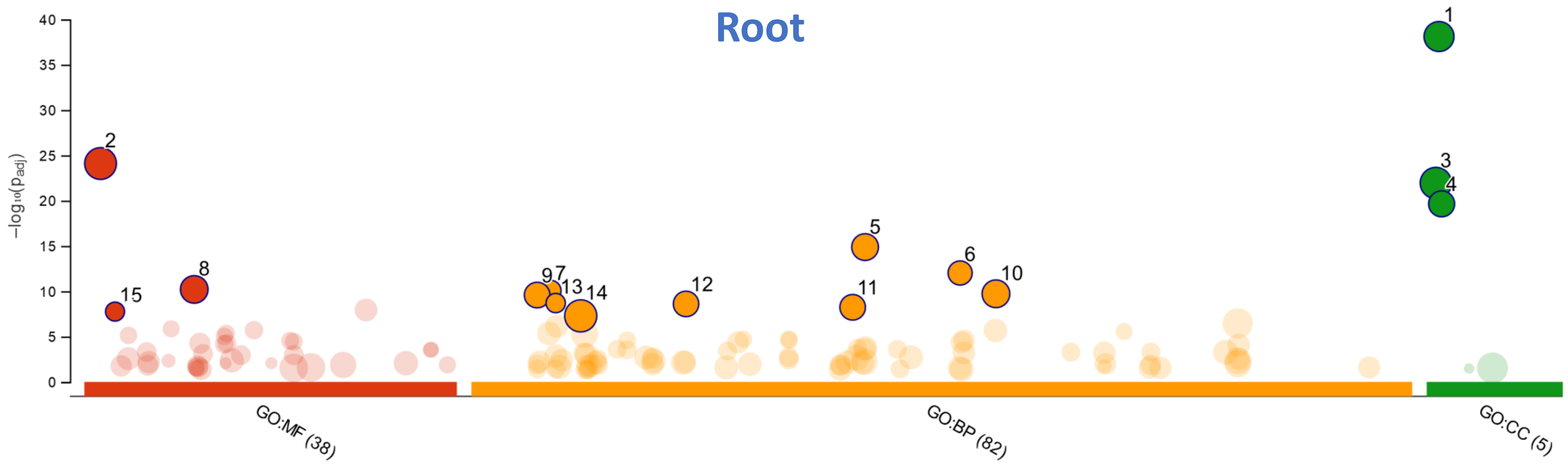

ID	Source	Term ...	Term Name	p_{adj} (query_1)
1	GO:CC	GO:0005737	cytoplasm	7.904×10^{-39}
2	GO:MF	GO:0003824	catalytic activity	8.095×10^{-25}
3	GO:CC	GO:0005622	intracellular	1.146×10^{-22}
4	GO:CC	GO:0005829	cytosol	2.279×10^{-20}
5	GO:BP	GO:0044281	small molecule metabolic process	1.336×10^{-15}
6	GO:BP	GO:0051186	cofactor metabolic process	9.696×10^{-13}
7	GO:BP	GO:0006575	cellular modified amino acid metabolic process	8.696×10^{-11}
8	GO:MF	GO:0016491	oxidoreductase activity	6.294×10^{-11}
9	GO:BP	GO:0006082	organic acid metabolic process	2.636×10^{-10}
10	GO:BP	GO:0055114	oxidation-reduction process	1.905×10^{-10}
11	GO:BP	GO:0043436	oxoacid metabolic process	5.869×10^{-9}
12	GO:BP	GO:0019752	carboxylic acid metabolic process	2.500×10^{-9}
13	GO:BP	GO:0006749	glutathione metabolic process	2.015×10^{-9}
14	GO:BP	GO:0008152	metabolic process	5.187×10^{-8}
15	GO:MF	GO:0004364	glutathione transferase activity	1.761×10^{-8}

version e99_eg46_p14_f929183
date 14.5.2020, 16:01:02
organism mtruncatula

g:Profiler

Figure S1d. GO enrichment analysis (g:Profiler) of the specific root proteome.

> query_1

CSRP

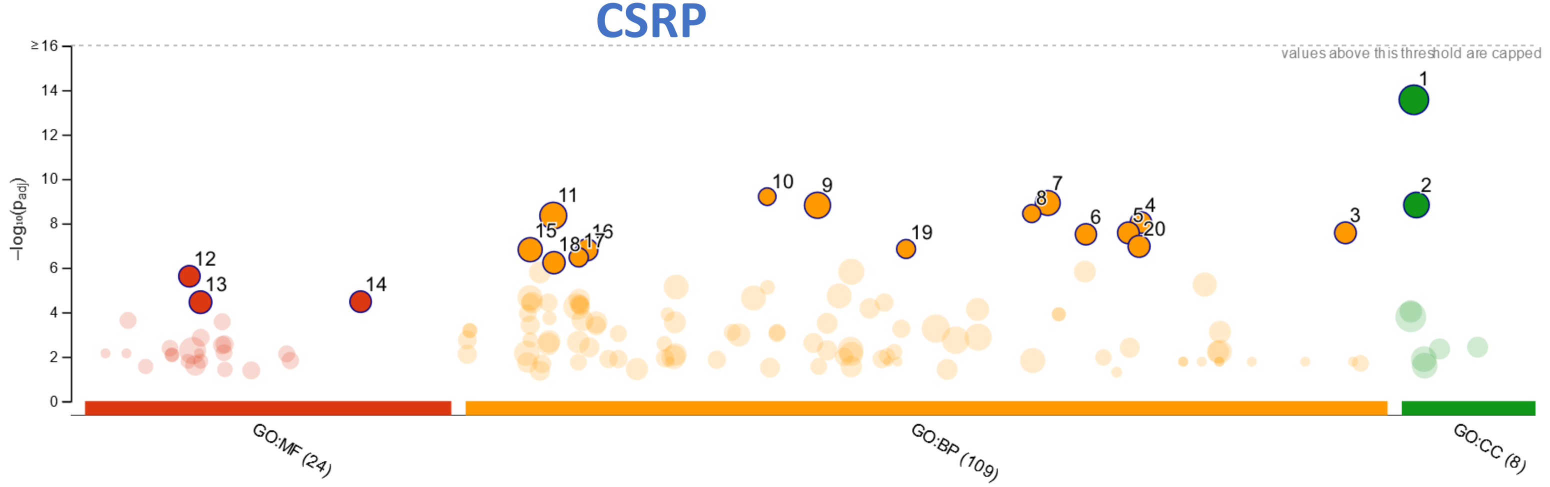

ID	Source	Term ...	Term Name	p_{adj} (query_1)
1	GO:CC	GO:0005737	cytoplasm	2.739×10^{-14}
2	GO:CC	GO:0005829	cytosol	1.490×10^{-9}
3	GO:BP	GO:1990748	cellular detoxification	2.668×10^{-8}
4	GO:BP	GO:0098869	cellular oxidant detoxification	9.150×10^{-9}
5	GO:BP	GO:0097237	cellular response to toxic substance	2.668×10^{-8}
6	GO:BP	GO:0072593	reactive oxygen species metabolic process	3.100×10^{-8}
7	GO:BP	GO:0070887	cellular response to chemical stimulus	1.207×10^{-9}
8	GO:BP	GO:0062197	cellular response to chemical stress	3.630×10^{-9}
9	GO:BP	GO:0042221	response to chemical	1.534×10^{-9}
10	GO:BP	GO:0034599	cellular response to oxidative stress	6.275×10^{-10}
11	GO:BP	GO:0006950	response to stress	4.526×10^{-9}
12	GO:MF	GO:0016209	antioxidant activity	2.405×10^{-6}
13	GO:MF	GO:0016853	isomerase activity	3.529×10^{-5}
14	GO:MF	GO:0051082	unfolded protein binding	3.345×10^{-5}
15	GO:BP	GO:0006091	generation of precursor metabolites and energy	1.530×10^{-7}
16	GO:BP	GO:0009636	response to toxic substance	1.548×10^{-7}
17	GO:BP	GO:0009132	nucleoside diphosphate metabolic process	3.397×10^{-7}
18	GO:BP	GO:0006979	response to oxidative stress	5.877×10^{-7}
19	GO:BP	GO:0046939	nucleotide phosphorylation	1.424×10^{-7}
20	GO:BP	GO:0098754	detoxification	1.081×10^{-7}

version e100_eg47_p14_7733820
date 10.9.2020, 13:21:15
organism mtruncatula

g:Profiler

Figure S1e. GO enrichment analysis (g:Profiler) of all Core Stress Response Proteins (Table S2).
