# Supplementary material for: Phloem Sap Proteins Are Part of a Core Stress Responsive Proteome Involved in Drought Stress Adjustment
Source: Front Plant Sci. 2021 Feb 2;12:625224. doi: 10.3389/fpls.2021.625224 (PMC7884324; doi:10.3389/fpls.2021.625224)
Supplement: Supplementary file 2 [file Data_Sheet_2.PDF]

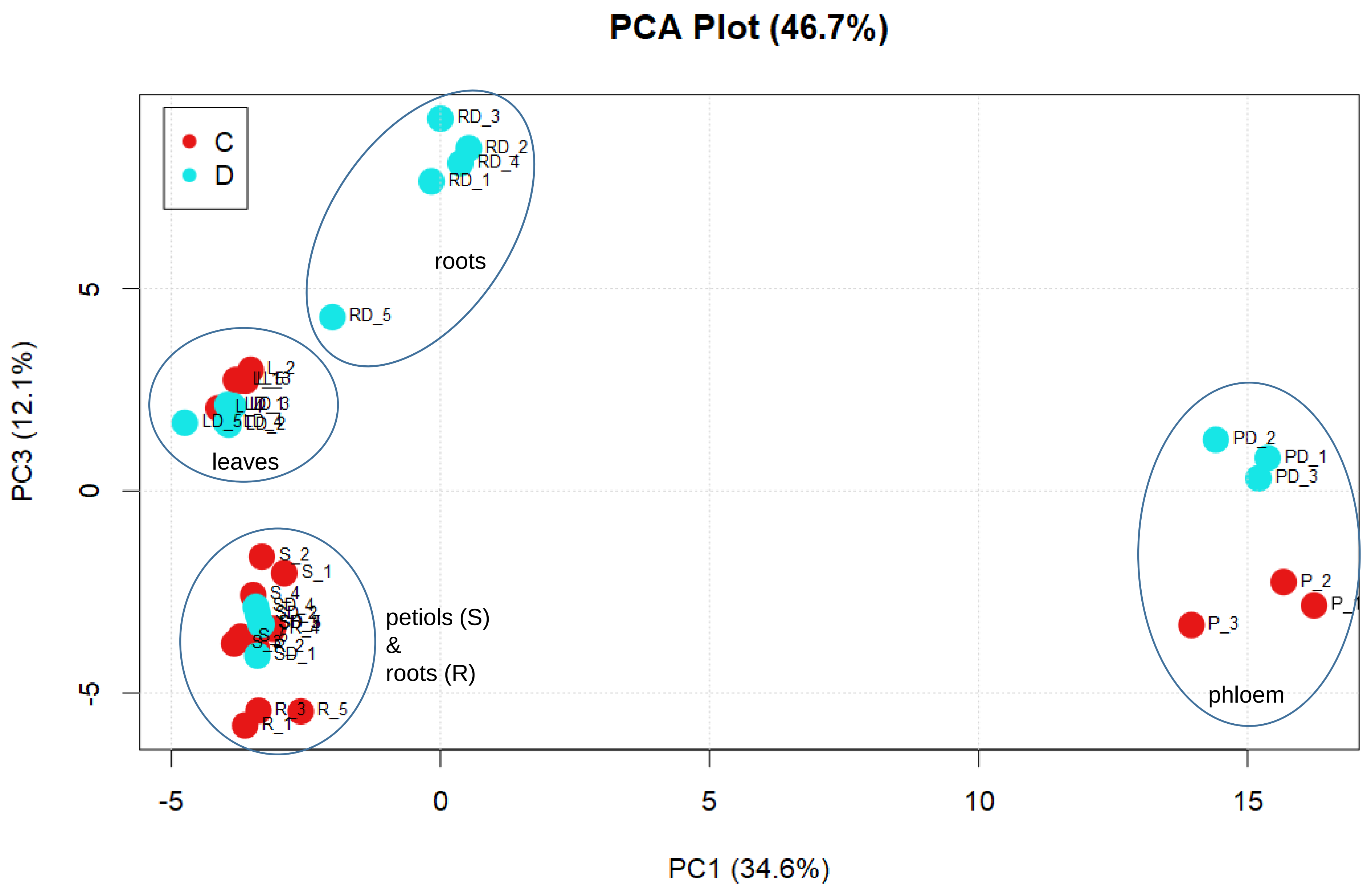

Figure S2. Principle component analysis of the Core Stress Responsive Proteome (Table S2). Highest loadings of PC1 (separation of phloem from other tissues) can be found in Table S2.
